# Artificial Sweeteners: History and New Concepts on Inflammation

**DOI:** 10.3389/fnut.2021.746247

**Published:** 2021-09-24

**Authors:** Abigail Raffner Basson, Alexander Rodriguez-Palacios, Fabio Cominelli

**Affiliations:** ^1^Division of Gastroenterology and Liver Diseases, Case Western Reserve University School of Medicine, Cleveland, OH, United States; ^2^Digestive Health Research Institute, University Hospitals Cleveland Medical Center, Cleveland, OH, United States; ^3^Mouse Models, Silvio O'Conte Cleveland Digestive Diseases Research Core Center, Cleveland, OH, United States; ^4^Germ-Free and Gut Microbiome Core, Digestive Health Research Institute, Case Western Reserve University, Cleveland, OH, United States

**Keywords:** artificial sweeteners, cytokines, inflammation, immunoregulation, Crohn's disease, ulcerative colitis

## Abstract

Since the introduction of artificial sweeteners (AS) to the North American market in the 1950s, a growing number of epidemiological and animal studies have suggested that AS may induce changes in gut bacteria and gut wall immune reactivity, which could negatively affect individuals with or susceptible to chronic inflammatory conditions such as inflammatory bowel disease (IBD), a disorder that has been growing exponentially in westernized countries. This review summarizes the history of current FDA-approved AS and their chemical composition, metabolism, and bacterial utilization, and provides a scoping overview of the disease mechanisms associated with the induction or prevention of inflammation in IBD. We provide a general outlook on areas that have been both largely and scarcely studied, emerging concepts using silica, and describe the effects of AS on acute and chronic forms of intestinal inflammation.

## Introduction

While sweet taste is one of the most desired flavors to mankind, it has been known for many years that excessive sugar consumption has adverse health effects. Artificial sweeteners (AS), also known as “non-nutritive” sweeteners, are agents that have a sweetening intensity higher than that of caloric/“nutritive” sweeteners (e.g., sucrose). Being ~200–20,000 times more potent than sucrose, AS are mainly used as a strategy to reduce the caloric/sugar content of foods.

While the Food and Drug Administration (FDA) deems AS to be safe, there is evidence that AS influence inflammation pathways (1–7). Owing to the potent sweetening effect of most AS and bitterness/lingering aftertaste, most commercial products contain a commercial proprietary blend of two or more AS, as well as ingredients to make AS more palatable. Commercial AS also contain fillers such as maltodextrin comprising 95–99% of the product to add weight and volume, or anti-caking agents such as silica. While these substances are considered innocuous in small quantities, several studies indicate that such fillers could also promote intestinal inflammation and changes in gut microbiota (8–11). Therefore, the variability in chemical composition makes the association of any potential AS to inflammation difficult to assess.

Preclinical animal studies indicate that some AS studied contribute to the development or worsening of gastrointestinal inflammation (1–7), while some are reported as having an anti-inflammatory effect (12). Findings are, however, controversial because of potential conflicts of interest, as AS manufacturers often sponsor professional organizations to author the studies.

Herein, we review the history of the FDA approval of AS and AS metabolism and bacterial utilization, and provide a scoping overview of the disease mechanisms associated with the induction or prevention of inflammation in models primarily relevant to chronic intestinal inflammation.

## History and Chemistry of Artificial Sweeteners

Artificial sweeteners first entered the food industry in the 1800s. However, since the 2000s, there has been an explosive increase in their consumption. In the United States, AS consumption is estimated to have increased by ~200% in children/adolescents and 54% in adults between 1999 and 2000, with ~25% of children and 41% of adults consuming AS at least once daily between 2009 and 2012 (13). Consumption of AS may, however, be more widespread because of their presence in “lower-calorie” food products as well as medications to improve palatability. The use of AS-altered diets is even listed in guidelines for the medical management of patients with inflammatory bowel disease (IBD) (14). Specifically, the British Society of Gastroenterology consensus guidelines recommend the use of Crusha flavoring, which has AS. Such additions to food and medications seem unjustified owing to rising evidence that AS affect inflammation pathways. AS have also been shown to exert physiological effects on glucose metabolism, appetite stimulation, and metabolic disease (e.g., type 2 diabetes; T2DM, metabolic syndrome, obesity) (3, 15–18).

**Figure 1:**
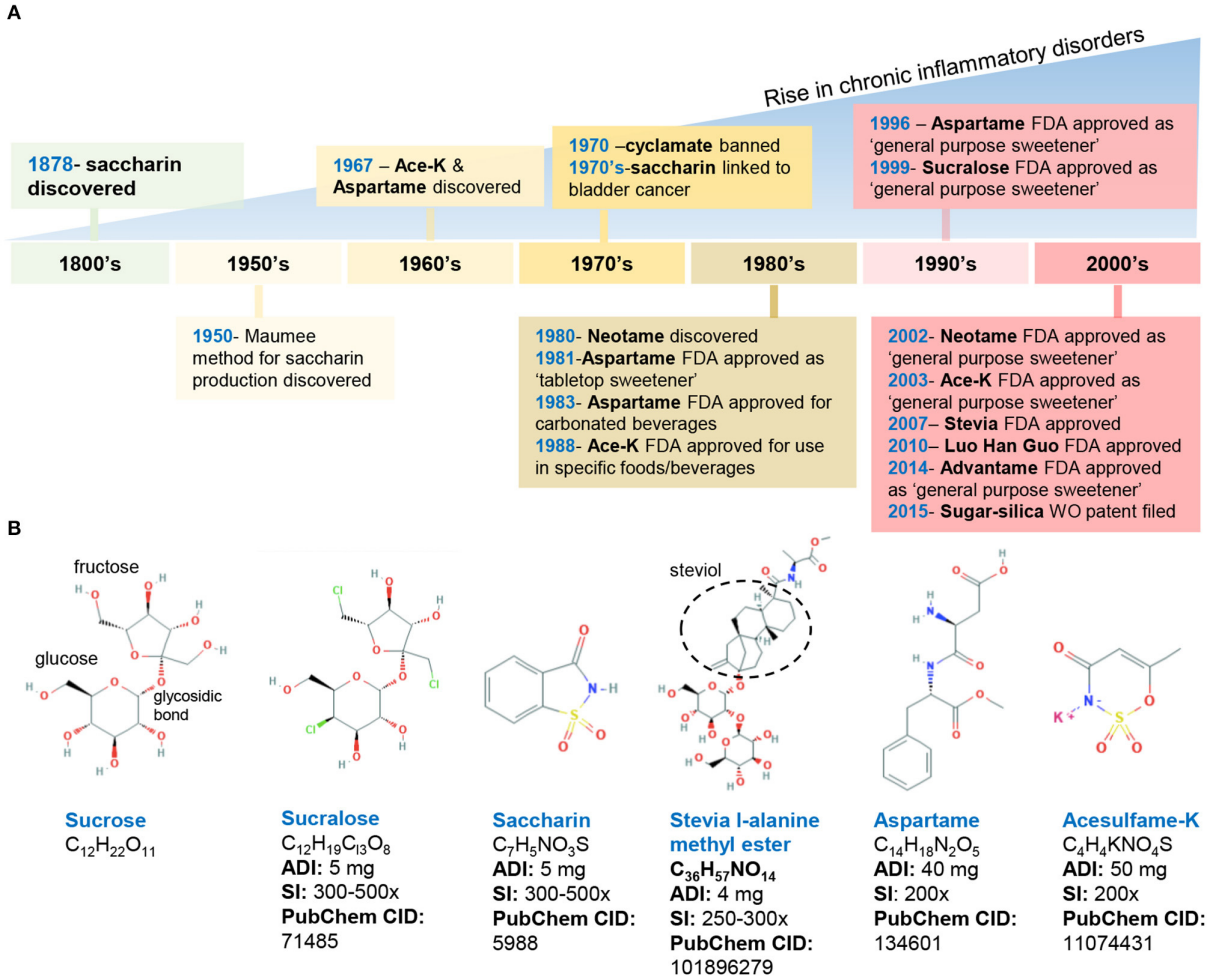
Historical timeline of artificial sweeteners approved by the FDA for use in the food industry. **(A)** timeline of FDA approved AS. **(B)** Chemical structure and characteristics of the commonly used AS. ADI, acceptable daily intake; SI, sweetness intensity (relative to sucrose); WO, world (2015/159156 A2). Images, public domain (https://pubchem.ncbi.nlm.nih.gov).

The introduction of AS by the food industry has been postulated to serve as an etiologic factor associated with the onset, progression, and severity of IBD (1, 19, 20). That is, the dramatic increase in IBD prevalence since the early 1990s in Canada, the USA, and England coincided with sucralose and saccharin becoming common ingredients in the food supply (Figure 1). Extrapolating from recent research, long-term consumption of sucralose appears to have significant adverse effects on the gut, namely, dysbiosis. Such disruption in the gut microbiota could be a major contributing factor to IBD that deserves further mechanistic studies.

In the United States, the FDA has approved eight AS, which include two AS of natural origin—stevia and monk fruit extract, and six synthetically derived AS, namely, aspartame, acesulfame potassium (Ace-K), neotame, saccharin, sucralose, and advantame. Below, we summarize the chemical composition and characteristics of FDA-approved AS (Figure 1).

Discovered in 1879, saccharin (1,2-benzisothiazol-3-one-1,1-dioxide) is FDA-approved for cooking or table use and for processed foods. Saccharin can be made by oxidizing o-toluene sulfonamide or phthalic anhydride; however, in 1950, an improved synthesis method was developed by the Maumee Chemical Company (Toledo, OH, United States), which involves diazotization of anthranilic acid to yield saccharin and is currently used in manufacturing (21). Because of its slight acidic taste, saccharin is often combined with other sweeteners such as aspartame and cyclamates. Of note, the FDA banned the use of cyclamate (E-952) in 1970 because of the detection of bladder tumors in rodents; however, cyclamate remains approved in over 50 countries (22–24).

Aspartame (N-L-α-aspartyl-L-phenylalanine1-methyl ester) is a methyl ester of a dipeptide composed of aspartic acid and L-phenylalanine that was discovered in 1967 (25). Due to its bitter aftertaste, aspartame is often combined with other sweeteners (Ace-K, cyclamates, sucralose). The sweetener is not heat-stable. Of note, aspartame in food with a pH higher than 6 can transform into diketopiperazine, a carcinogenic compound (26).

Acesulfame potassium (6-methyl-1,2,3-oxathiazine-4(3H)-one 2,2-dioxide) is the potassium salt of asulfame, a hydrophilic acidic cyclic sulfonamide that belongs to the oxathiazinonedioxide class (27). Similar to saccharin and cyclamate, Ace-K belongs to a chemical class associated with antimicrobial activity (28). Ace-K was accidently discovered in 1967 and subsequently studied on mice and dogs to determine its short- and long-term safety. Ace-K is heat-stable, although it is typically used in candies, beverages, and frozen desserts (25).

Sucralose (1,6-dichloro-1,6-dideoxy-β-D-fructofuranosyl-4-chloro-4-deoxy-α-D-galactopyranoside) is structurally similar to sucrose *via* the replacement of hydroxyl groups with chlorine in the 4, 1′, and 6′ positions (29). This AS is water soluble and heat-stable, and it has negligible effects on pH or viscosity (27).

Neotame N-[N-(3,3-dimethylbutyl)-l- aspartyl]-L-phenylalanine 1-methyl ester) was discovered in the 1980s and is obtained by the reductive alkylation of aspartame, which is converted into 3,3-dimethylbutraldehyde and thus, is structurally similar to aspartame (i.e., N-N-(3,3-dimethylbutyl-L-α-aspartyl-L-phenylalanine-l-methyl ester). Neotame is moderately heat-stable (25).

Advantame is the most recently approved synthetic AS, receiving the approval by the FDA as a general- purpose sweetener and flavor enhancer. Advantame is an N-substituted derivative of aspartame made from aspartame and vanillin; however, unlike aspartame, it can be consumed by individuals with phenylketonureia. Advantame is heat-stable (30).

The two FDA-approved naturally occurring AS include stevia leaf extract (steviol glycosides) and Luo Han Guo fruit extracts. Steviol glycosides (including 10 different glycosides) are sweet-tasting molecules derived from the *Stevia rebaudiana* plant (Ateracean family) native to Paraguay and Portugal. Four major and six less prevalent steviol glycosides have been discovered, of which stevioside (5–10%) and rebaudioside A (2–5%) are the most abundant followed by various rebaudiosides (B, C, D, F, M) (27, 30). All these Steviol glycosides have a central steviol structure but are conjugated with different sugar residues. The proposed beneficial properties of steviol glycosides are attributed to the compounds that comprise this mixture; however, the presence of which varies based on extraction and processing methods (30).

Luo Han Guo (*Siraitia grosvenorii* swingle) fruit extracts from monk fruit come from a plant native to southern China and contain varying levels of mogrosides (11-α-hydroxy-mogrosides), glycosylated cucurbitane-type teriterpenoids which account for the characteristic sweetness (31, 32).

While all AS share a sweet taste, each is a chemically distinct compound; thus, the pattern of response of gut microbes and their effects on the gastrointestinal tract in terms of how they are transported in the small and large intestines, metabolized and excreted, differ depending on the sweetener (27).

## Metabolism and Bacterial Utilization of Artificial Sweeteners

**Figure 2:**
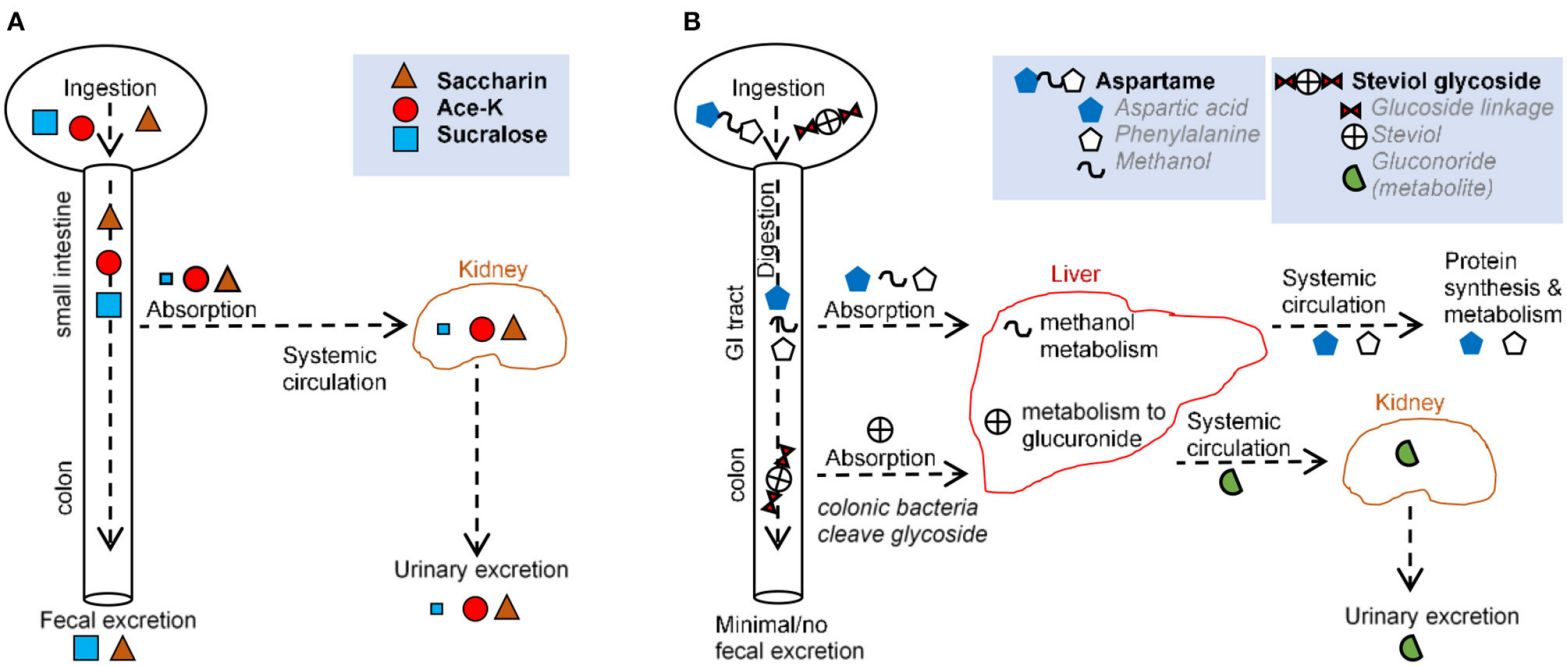
Comparison of main routes of absorption, digestion, metabolism, and excretion of representative AS **(A,B)** illustrate the absorption, digestion, metabolism, and excretion for **(A)** saccharin, acesulfame-potassium, sucralose, and **(B)** aspartame and steviol glycoside.

The proposed advantage of most non-nutritive AS is that following ingestion they are not metabolized (33, 34). Some AS are known to be compound molecules that are amenable for degradation by bacteria. Some sweeteners, i.e., sucralose, were originally thought not to be metabolizable. However, mass spectrometry studies showed that the spectral profile of the molecule recovered from feces is structurally different from that which was ingested, indicating that such AS can be metabolized in the gut, possibly by bacteria. In other cases, AS, such as stevia, are broken down into simpler molecules that can be metabolized by the host or bacteria (27). In such scenario, the AS core molecule, steviol, is absorbed systemically, and then excreted in urine (27). While AS are known to modulate the gut microbiota, little is known regarding their effect on viruses and fungi in the gut. The section below and Figure 2 provide an overview of absorption-excretion patterns for representative AS and the influence of gut bacteria.

Over 85% of saccharin is systemically absorbed in the small intestine. Following absorption from the gastrointestinal tract (GIT), saccharin binds to plasma proteins and is distributed throughout the body. Saccharin is then eliminated unchanged primarily *via* urination, and the remainder is excreted *via* defecation (27, 35). Saccharin absorption varies based on stomach pH, with low stomach pH (e.g., in humans) increasing absorption and higher pH (e.g., in mice and rats) decreasing absorption (27). *In vitro*, saccharin exerts a dose-dependent effect on bacteria isolated from the oral cavity (36) and from the GIT (37, 38), with one *in vitro* model demonstrating an increase in *Bifidobacteria* and a decrease in *Firmicutes* (39). In animal studies, saccharin exhibited bacteriostatic and microbiome-modulating properties (3, 37, 40) favoring anaerobes (37), consistent with the shift from obligate anaerobes to facultative anaerobes and aerobes reported in IBD (41). Saccharin-induced dysbiosis has led to alterations in metabolic pathways linked to glucose tolerance (3).

The aspartame dipeptide is hydrolyzed by esterases and peptidases in the gut and broken down into amino acids, aspartate, phenylalanine, and methanol. These compounds are absorbed in the duodenum and jejunum and metabolized *via* their usual metabolic pathways (35). Thus, negligible amounts of the intact molecule reach general circulation (27, 42, 43). Notably, the same degradation products (aspartate, phenylalanine, methanol) are present at higher concentrations following the ingestion of fruits, vegetables, meats, and dairy. This is important because *in vitro* studies, in which aspartame has been administered, bypass the process of digestion and thus, do not provide biologically plausible scenarios. While many studies have described aspartame as inert, some have suggested some form of hypersensitivity that occurs in a dose-dependent manner (30). *In vivo*, aspartame supplementation lowered food intake and body weight gain, with elevated fasting glucose levels and impaired insulin-stimulated glucose disposal, independent of body weight (44).

Acesulfame potassium is almost completely absorbed as an intact molecule in the small intestine and distributed to the blood and different tissues (27, 45). Ace-K is excreted in the urine within 24 h with <1% eliminated in feces (27, 46). Although a negligible amount of Ace-K reaches the fecal or colonic bacteria (27, 46, 47), animal studies report Ace-K-induced shifts in the composition of gut microbiota (48, 49), with consumption during pregnancy is linked to metabolic/microbiome alterations in progeny (50). Ace-K has also been shown to inhibit glucose fermentation by intestinal bacteria (51, 52), and alter bacterial genes involved in energy metabolism (carbohydrate absorption, metabolism, fermentation pathways) (48). Such microbial alterations were correlated with increase in body weight (48). In healthy adult humans, consumption of Ace-K and aspartate resulted in an overall decrease in bacterial diversity (53).

Sucralose passes primarily unabsorbed through the GI tract and is recovered structurally unchanged in feces (70–90%) and urine (14.5%) (27, 33). Mass spectrometry data indicate that some sucralose molecules are chemically altered as they pass through the rodent GIT (54, 55), suggesting metabolism and secondary metabolite production. The small proportion of sucralose that is absorbed is eliminated mostly unchanged in the urine, although two glucuonide conjugates of sucralose have been detected (accounting for ~2.6 of the administered dose %) following a single oral dose in healthy volunteers (33). Intriguingly, increases in body weight, not attributed to food intake, have been reported in mice administered with Splenda in “low” doses (100 mg/kg bw/day) but not “high” doses (300, 500, and 1,000 mg/kg) (56). Human, animal, and *in vitro* studies suggest that sucralose reduces bacterial growth (1, 56, 57), may selectively inhibit or promote bacterial growth (29), and promote intestinal dysbiosis (33, 49), consistent with the observation that bacteria in a culture do not utilize sucralose as a carbon source (58, 59). Sucralose may increase *Escherichia coli* antimicrobial resistance and mutation frequency to antimicrobials (60) and inhibit the bacterial physiology of differentiating filamentous cyanobacteria and polysaccharide sheath induction (61). Sucralose elicited strong bacteriostatic effects on *Streptococcus* species (29, 62) because of its ability to prevent sucrose (table sugar) absorption in most microorganisms (29). Mechanistically, sucralose inhibits bacterial invertase and sucrose permease, two enzymes unable to catalyze the hydrolysis or transmembrane transport of sucralose (29). It is possible that bacterial cells can internalize sucralose, making them susceptible to the chemical effects of the compound (63).

Neotameis is metabolized by esterase into de-esterified neotame and methanol, which are eliminated within 72 h *via* urine and feces (64, 65). About 50% of the absorbed neotame is eliminated in urine as de-esterified neotame, the remaining passes through the gut and is excreted in feces. A negligible by-product of neotame metabolism is methanol. Neotame, even at doses above the ADI, has not shown signs of toxicity (30).

Advantame is mostly converted to ANS9801-acid (de-esterified advantame) in the gut prior to absorption. Advantame that is absorbed intact is converted to ANS9801-acid in the plasma (66). Approximately 90% is excreted in feces and 6.2% in urine. In feces, more than half of ANS9801-acid is excreted as de-esterified aspartame, and the remainder is mostly excreted as N-(3-(3-hydroxy-4-methoxyphenyl)) propyl-L-aspartic acid [which may be further degraded to 3-(3-hydroxy-4-methoxyphenyl)-1-propylamine) and phenylalanine] (66, 67). In urine, 2.3% is excreted as de-esterified advantame, 1.9% as 5-(3-aminopropyl)-2-methoxyphenyl, and 1% as the aspartic acid analog (67).

Steviol glycosides cannot be hydrolyzed by upper GIT enzymes or acids (68). In the colon, *Bacteroides* are the only bacteria capable of hydrolyzing steviol glycosides to steviol (69–72). Steviol is resistant to bacterial degradation. While some is excreted in feces, the majority is absorbed and conjugated with glucuronic acid in the liver (72, 73). Steviol glucuronide is then primarily eliminated in human urine (73–75). Of note, colonic epithelial cell lines (e.g., Caco-2) take up steviol but not stevioside, suggesting differences in the biological function/relevance between steviol and stevioside (76). *In vitro*, rebaudioside A has been shown to inhibit aerobic bacteria weakly, in particular coliforms, whereas stevioside inhibits anaerobic bacteria weakly (72). Overall, stevia appears to modify the gut microbiota (77).

Mogrosides are mostly degraded in the colon by digestive enzymes and gut microbiota, which cleave the glucose molecules as a source of energy (78). The remaining mogrol and its mono- and diglucosides are then excreted in feces. In rats, a trace amount of mogrol and its monoglucoside were identified in the portal blood as sulfates and/or glucorinide conjugates following a single ingestion (79).

To add volume to AS, marketed products often contain maltodextrin (MDX) (or others such as silica or calcium silicate) as a filler (weight and volume). As a novel strategy to make the consumption of sugar “safer,” silica has been used recently as a scaffold for sugar to lower the total caloric content of a product, making this type of combination an artificial alternative to AS (80).

A caveat of studying proprietary AS mixtures (e.g., products containing several AS and/or fillers) is that multiple ingredients may interact with one another, rendering the studies primarily relative to the multi-ingredient product tested and generating information associated with metabolism or inflammation that is difficult to trace to the specific AS ingredient that we recently highlighted as a strategy to help draw guidelines on dietary recommendations for patients with IBD (81).

### Local and Systemic Bacterial Metabolites

Several animal studies have demonstrated AS-induced metagenomics alterations in bacterial genes and the subsequent bacterial byproducts following AS supplementation. Such alterations could increase or attenuate the risk of inflammation in the host, as these mediators could translocate into circulation and elicit an anti- or pro-inflammatory response.

#### Tryptophan Metabolism

Tryptophan metabolism *via* the kynurenine pathways plays an important role in inflammation and immunity (82–84). Tryptophan metabolism alterations, following chronic consumption of sucralose (5) or saccharin (4) at levels equivalent to FDA-approved human ADI, have been reported in C57BL/6J mouse gut microbiome. Specifically, in feces, sucralose altered four compounds that modulate inflammation; L-tryptophan, quinolinic acid, 2-aminomuconic acid (all increased), and kynurenic acid (decreased) (5). Of note, quinolinic acid has been reported as pro-inflammatory, whereas kynurenic acid is anti-inflammatory and neuroprotective (85). Metabolites of tyrosine metabolism were also altered, with increases in L-tyrosine and decreases in p-hydroxyphenylacetic acid and cinnamic acid (5); the latter is known to suppress the production of reactive oxygen species (86).

Similar to those treated with sucralose, saccharin-treated mice exhibited significant increases in quinolinic acid (4), and had decreased equol production (4), a daidzein metabolite shown to inhibit lipopolysaccharide (LPS)-induced oxidative stress in macrophages and suppress inflammatory response in mice (87–89). Notably, diadzein significantly increased in the saccharin-treated mice, indicating that saccharin reduced the growth or decreased the enzymatic activity of metabolizing bacteria (4), which was consistent with the observed decreased abundance of *Adlercreutzia*, a genus that contains equol-producing bacteria (4, 90).

#### Short Chain Fatty Acid Synthesis

Short chain fatty acids, primarily acetate, proprionate, and butyrate, are produced during the bacterial fermentation of dietary fibers in the colon. Short chain fatty acids act as anti-inflammatory metabolites in the gut, particularly *via* regulation of T-regulatory cells. In IBD, SCFAs are typically reduced in gut mucosa and feces of patients with IBD patients (91).

Animal studies have demonstrated AS-induced alterations in SCFA synthesis. *In vivo*, sucralose increased the number of SCFA-related genes, especially in the presence of a high-fat (saturated) diet (92). In another study, aspartame elevated circulating SCFAs, particularly propionate (44), a highly gluconeogenic substrate that has been suggested to explain the negative effects of aspartame on insulin tolerance. Neotame changed the composition of gut microbiota (it decreased *Firmicutes* and increased *Bacteroidetes*), reduced alpha-diversity, and decreased bacterial genes involved in butyrate synthesis (93). Other studies have shown changes in gut microbial butyrate and pyruvate production as a result of Ace-K (48). *In vitro*, steviol incubation in the BISI-phase-2 system, a model that simulates human intestinal microbial environment, reduced *Bifidobacteria* and levels of ammonia, increased pH, and negatively influenced SCFA ratio (39). In rats, low-dose *Stevia rebausiana* reduced dopamine transporter mRNA and nucleus accumbens tyrosine hydroxylase levels, and increased levels of SCFAs acetate and valerate (77). Such findings indicate that AS interact with the gut and peripheral tissues/immune responses *via* bacterial SCFA production.

#### Bacterial Genes Involved in Energy Metabolism

Acesulfame potassium has been shown to exert gender-specific effects on fecal metabolite profiles in CD1 mice (48). Specifically, Ace-K-treated males had increased body weight gain (vs. females), and exhibited increased *Bacteroides* with significant changes in *Anaerostipes* and *Sutterella*. Corresponding to increased *Bacteroides*, Ace-K promoted genes involved in carbohydrate absorption, metabolism, and fermentation pathways in male mice. In contrast, Ace-k-treated females exhibited a decrease in *Lactobacillus, Clostridium*, unassigned *Ruminococcaceae* genus, and *Oxalobacteraceae* genus, and increased the abundance of *Mucispirillum*, with a decrease in many of the genes involved in energy metabolism and carbohydrate absorption or transport such as lactic acid, succinic acid, and 2-Oleoylglycerol. By comparison, males had significantly higher concentrations of pyruvic acid, a metabolite central to energy metabolism (48). The mechanism of action underpinning these gender differences is unclear, since most rodent studies house several animals per cage, and it is unknown to what extent cage effects may influence data in gender-specific diet studies (94).

#### Effect on Intestinal Epithelial Cells

Artificial sweeteners can directly modulate the composition and function of the microbiota, although the mechanism of action by which AS modify the gut microbiota is not fully understood. One possible mechanism of action is the secretion of defensins, which are known to modulate the gut microbiome. Alternatively, AS can modulate epithelial cells that are in close contact with the lumen. In Caco-2 cells, which are found on the wall of the intestine, AS (saccharin, sucralose, and aspartame) administered at physiological concentrations easily achieved by diet (100 uM) differentially increased biofilm formation, and the bacterial ability to adhere to, invade, and kill mammalian gut epithelial cells (95). Additionally, all the three sweeteners caused gut bacteria pathogens *E. coli* and *Enterococcus faecalis* to invade Caco-2 cells, with the exception of saccharin, which had no significant effect on *E. coli* invasion (95).

### Metabolic and Inflammatory Effects of AS Depend on the Diet

The extent to which specific bacteria are selectively modified by AS reflects diet composition, for instance, the presence of dietary saturated fat (92). In Wistar rats fed either with a high-fat diet (HFD) or a standard rodent diet supplemented with sucralose, steviol glycoside, or a caloric sweetener (e.g., sucrose), gut microbiota were differentially modified by both the type of sweetener and the fat content of the diet, explaining up to 48.5% of microbiota variation (92). While steviol glycoside resulted in the lowest number of LPS synthesis genes and produced the highest serum IL-10 compared with other AS, mice fed either with steviol + HFD, sucrose + HFD, or sucralose + HFD had the highest number of LPS synthesis genes. Thus, the effect of an AS depends on the diet composition, and this modulates the effects of AS on inflammation (92).

The same concept applies to food intake (gm/day consumed), for which studies have reported AS-induced alterations, either increasing or decreasing caloric intake (96). This is important, since the effects on colonic microbiota, as reported by studies, may be attributed to changes in food intake, rather than the actual AS tested. Overall, an AS-supplemented diet may modify bacterial functionality with subsequent by-products that directly or indirectly trigger/modify inflammation locally in the gut or systemically in other organs, for example, the liver.

## Host Genetics Mediates Artificial Sweetener Effects

Several lines of evidence suggest that the effect of AS on inflammation and severity of response to AS relative to gut microbial changes (or other outcomes, i.e., glucose intolerance) depends on the genetic susceptibility of the host. For instance, in a recent study, a 6-week intake of Splenda, supplemented at the maximum dose recommended by the FDA (3.5 mg/ml), had significant outcomes on ileitis (increased myeloperoxidase, MPO, activity, penetration of gut bacteria in gut wall) in mice prone to IBD (SAMP1/YitFc; SAMP), but not in healthy control AKR/J mice despite changes being observed in the gut microbiome (1). This study illustrates that changes in the gut microbiota may reflect AS consumption, but such changes are not necessarily correlated with IBD unless the consumer has genetic susceptibility. Similar discrepancies in the effect of AS relative to different mouse lines have been reported (97). Specifically, Splenda and stevia-treated CD1 mice had higher percentage of lymphocytes in Peyer's patches compared with Balb/c mice (97), whereas lymphocyte proportions were increased in sucrose-treated Balb/c mice but reduced in CD1 mice (97).

Such variability across lines has also been reported for glucose tolerance. Overall, diabetic mouse models have demonstrated that AS exert anti-hyperglycemic effects in rodents (98, 99). However, similar studies using “healthy” mouse lines (e.g., Balb/c, C57BL/6J, CD1) that were supplemented with stevia or sucralose have revealed AS-induced glucose intolerance, insulin resistance, and significantly elevated HbA1clevels (a marker reflecting over 3 months of average blood glucose levels) (2, 3, 100, 101).

Many studies have investigated the effects of AS *in vitro*, using multiple available human cell lines. In human cancer cell lines, differences in the effect of steviol in attenuating the release of TNFα-mediated IL-8 (an important mediator of the innate immune reaction) were observed, with steviol having the most robust effect on the T84 cell line compared with that of the Caco-2 and HT29 cells (102). It is important to highlight that *in vitro* data using human cell lines introduce a confounder due to genetic differences that exist from the individual source of the cell lines. Moreover, every cell line has a different epigenetic make-up, is already specialized, and exhibits a particular phenotype.

Further exemplifying the role of genetics and host gut microbiota composition, Suez et al. showed that AS consumption (saccharin) induced dysbiosis and glucose intolerance in some (“responders”) but not all healthy volunteers (“non-responders”) (3). Moreover, the effects on glucose intolerance were transferrable *via* the microbiota to germ-free mice, but only from donors identified as “responders” (3). The findings highlight not only the role of AS-induced alterations to the gut microbiota in the development of metabolic alterations, but also the effect of host genetics, which is recognized for its modest role in shaping the host microbiome (103).

## Artificial Sweeteners on Digestive Inflammation Outcomes

Studies focused on the inflammatory potential of stevia have yielded inconsistent results. Discrepancies may reflect the type/dosage/purity of the tested compound and host genetics (104), as discussed above. For instance, stevioside (50, 100 mg/kg body weight) for 7 days prior to dextran sodium sulfate (DSS) colitis induction, a widely used model of IBD that exhibits symptoms similar to that of ulcerative colitis, significantly lowered clinical signs of colitis (body weight, disease activity, colon length, histology) in Balb/c mice (12). In contrast, the long-term administration of the commercial product SweetLeaf (4.20 mg/ml stevia), a mixture of stevia leaf extract, silica and inulin, for 18 weeks significantly elevated liver enzyme levels (alanine aminotransferase, ALT; aspartate aminotransferase, AST), kidney function enzymes (urea, creatinine), and histological inflammation in Balb/c albino mice (2). Since the gut microbiota may impact the overall effect, it is important to note that microbiome composition was not evaluated in either study (2, 12).

The gut-liver axis refers to the bidirectional relationship, which stems from the integration of signals derived from diet, genetics, and environmental factors, between the gastrointestinal tract and its microbiota, and the liver,. A number of animal studies have reported saccharin to exert hepatotoxic effects (4, 105), wherein short-term administration resulted in transaminitis (elevation in ALT, AST, and ALP) (106) and long-term exposure promoted hepatic inflammation (4). Saccharin also promoted the overexpression of key oncogenes such as H-ras while reducing expression p27, a tumor suppressor gene (107). Saccharin has also been reported to promote gut taxa previously associated with pro-inflammatory effects (3, 4, 45, 90, 108–111).

The long-term consumption of aspartame has been shown to induce liver degeneration, necrosis, fibrosis, and mononuclear cell infiltration, mediated in part through an imbalance in redox homeostasis and adipocytokine dysregulation in rodents (107, 112–114). Prolonged aspartame consumption increases methanol and its metabolites, which are associated with oxidative stress. In a folate-deficient mouse model (which mimics human methanol metabolism), aspartame-mediated liver damage resulted from aspartame-derived metabolites (115).

In rodents, sucralose elicited adverse effects on gut tissue/barrier *via* the alteration of host microbiota and related metabolites and impaired inactivation of digestive proteases. In Sprague-Dawley rats, 12-week sucralose (Splenda; sucralose + maltodextrin) administration induced inflammatory lymphocyte infiltration, epithelial scarring, and mild depletion of goblet cells (56). As mentioned, in SAMP mice (genetically susceptible to IBD), Splenda (3.5 mg/mL, 6 weeks) resulted in significant deleterious outcomes on ileitis, an effect not observed in the healthy control AKR/J line (1). Other sucralose-containing commercial products, such as Sweetal (sucralose + sorbitol), significantly elevated liver enzyme levels (ALT, AST) and kidney function enzymes (urea, creatinine) in Balb/c albino mice after 18 weeks (administered 5 h/day in drinking water; 5.2 mg/ml sucralose) (2). Similar *in vivo* pro-inflammatory effects [hepatic mediators (5), severity of chemically-induced colitis (6, 57)] following pure sucralose supplementation have also been reported (5, 6, 57).

The current data regarding Ace-K on inflammation, hepatic or gut, are limited. Ace-K-treated C57BL/6J mice (150 mg/kg, 0.06%w/v, 6 weeks) exhibited increased lymphocyte recruitment to intestinal microvessels, expression of inflammatory cytokines (TNFα, IFNγ, IL1β), and endothelial/trafficking adhesion molecules (ICAM-1, VCAM-1, MAdCAM-1) (116). Not much is known about the effect of neotame on inflammatory outcomes. Long-term consumption has been associated with low body weight and body weight gain, albeit this was allometrically consistent with changes in food consumption (117).

### Bacterial Lipopolysaccharide, Flagella, and Toxins

The impact of different AS on the composition of the gut microbiota has been extensively reviewed elsewhere (15, 35). LPS, flagella, and fimbriae are bacterial attributes that can, in parallel, trigger several similar inflammatory mediators and induce intestinal inflammation (118, 119), and promote disruptions in the integrity of the gut barrier (120, 121). LPS is an endotoxin from the outer membrane of the gram-negative bacterial cell wall, which increases intestinal permeability and stimulates the monocyte and macrophage production of inflammatory mediators associated with IBD, such as TNFα, IL-1β, IL-6, and reactive free radical nitric oxide (NO) (3, 92, 122, 123). In general, during homeostasis, the host is exposed to low levels of LPS, but the excessive production and release of LPS signs of inflammation may occur *via* the expansion of gram-negative bacteria, which can be affected by AS.

Increases in LPS and bacterial pathways of LPS biosynthesis have been reported for most AS, such as stevia (2, 92), sucralose (2, 5, 92), saccharin (3, 4), and Ace-K (48). In C57BL/6J mice, saccharin (0.3 mg/ml—eq. ADI, 6 months) enriched six LPS biosynthesis orthologs a flagellar assembly ortholog, and six multidrug resistance, 11 fimbrial, and 23 bacterial toxin orthologs (4). Saccharin also decreased anti-inflammatory compounds such as palmitoleoyl ethanolamide (PEA), N,N-Dimethylsphingosine and linoleoyl ethanolamide (LEA) (4), and the latter was reported to reduce LPS-induced inflammation in macrophages (88). In another long-term study, sucralose enriched bacterial genes related to LPS synthesis and multiple genes associated with flagella protein synthesis, fimbriae synthesis, and bacterial toxin genes such as shiga toxin subunits and toxic shock syndrome in C57BL/6J mice, (5). In CD1 mice, Ave-K treatment (37.5 mg/kg/day, 4 weeks) significantly increased multiple genes encoding flagella components (FlgA, FlgH, FlgI, FliL proteins) and two genes participating in LPS biosynthesis and LPS-export genes, namely, glycosyltransferase and UDP-perosamine 4-acetyltransferase (48). Ace-K also increased bacterial toxin synthesis gene and thiol-activated cytolysis.

Lipopolysaccharide is a phosphorylated glycolipid and its structural similarity to some host-derived lipids (e.g., phosphatidic acid, ceramide) dictates that LPS and cholesterol share common trafficking and disposal pathways *in vivo* (124). High-density lipoproteins (HDLs) are also known to neutralize LPS *via* the presence of LPS-binding protein in HDLs (124). Of note, significant elevations in LPS have been accompanied by reductions in circulating HDL following long-term sucralose or stevia supplementation in BALB/c albino mice (2).

In other cases, while the effect of AS on LPS was not evaluated, it could be presumed that the expansion of gram-negative bacteria (outer membrane composed of LPS) could be associated with greater inflammation susceptibility. For example, saccharin, sucralose, aspartame, and Ace-K were shown to promote pro-inflammatory, gram-negative taxa such as *Bacteroidetes* (5, 48) *and Enterobacteriaceae* (44). On the other hand, some studies have found Splenda to increase *Proteobacteria* abundance after 6 weeks of supplementation (1), while some have reported dose-dependent reductions in total anaerobes after 12-weeks, such as *Bacteroides*, but no effect on *Enterobacteriaceae* has been reported (56). At higher doses (> 100 mg/kg/d), Splenda significantly reduced the numbers of total aerobes, an effect that perpetuated 12-weeks after Splenda cessation, suggesting that anaerobes do not recover following long-term sucralose supplementation (56).

Less is known on the impact of advantame or neotame administration on composition of the gut microbiota. In one study, neotame treatment (4 weeks) enriched *Bacteroidetes* abundance, amino acid metabolism, LPS biosynthesis, antibiotic biosynthesis, and folate biosynthesis pathways (93).

### Bile Acid Metabolism

Colonic bacteria are able to convert primary bile acids into secondary bile acids *via* deconjugation, dehydroxlation, and dehydrogenation. Bile acids facilitate the absorption of fat and fat-soluble vitamins, maintain cholesterol homeostasis, and serve as signaling molecules *via* binding to the nuclear receptor FXR and TGR5, a G-protein coupled receptor associated with metabolic regulation, which includes inflammatory response, cancer and liver regeneration (125).

Several studies have demonstrated AS-induced changes in bile acid homeostasis and metabolism, although results have varied. For instance, sucralose, but not Ace-K, at dosages equivalent to the maximum ADI (15 mg/kg/day in drinking water), increased hepatic cholesterol and cholic acid levels, and the ratio of secondary/primary bile acids after 8 weeks in C57BL/6J mice (49). By comparison, daily gavage with Ace-K (37.5 mg/kg/day) for 4 weeks increased fecal cholic acid but decreased deoxycholic acid in CD1 mice (48).

Sucralose has altered the bile acid profile *in vivo*. In C57BL/6J mice, 6-month sucralose supplementation (1.5 mg/ml in drinking water) increased 3-oxo-4,6choladienoic acid and reduced 3a,7b,12a-trihydroxyoxocholanyl-glycine, 3b,7a-dihydroxy-5-cholestenoate, and lithocholic acid (5). In another study, 6-week sucralose supplementation at the same dosage in C57BL/6 mice prior to colitis induction reduced the deactivation of digestive proteases mediated by deconjugated bilirubin (6). Other studies on rats have shown that sucralose or saccharin (6 weeks) significantly increased fecal chymotrypsin and trypsin, and decreased β-glucuronidase, an enzyme required for the deconjugation of conjugated bilirubin (57, 126).

### Intestinal Permeability and Inflammation Pathways

#### Tight Junction Proteins

Few studies have investigated the effect of AS on tight junction protein integrity. The intestinal barrier and epithelial cell homeostasis are maintained by an equilibrium between cell proliferation and death, and the paracellular space, which is modulated by tight junction proteins (primarily occludins, claudins, zonulin-1, junctional-adhesion molecules) that control the movement and circulation of intestinal contents (water, nutrients, electrolytes) across the epithelium into the lamina propria (127).

*In vitro*, the administration of aspartame to Caco-2 cells induced ROS production leading to increased permeability and internalization of claudin-3 (7). These effects were reversed by the overexpression of claudin-3, indicating its key role in the regulation of AS-induced intestinal permeability (7). A similar study showed that in gut epithelial cells, AS exposure increased apoptosis and permeability across the intestinal epithelium (7). *In vivo*, sucralose supplementation (1.5% in water) decreased colonic occludin abundance, an effect that was exacerbated when the supplementation was combined with a high-fat diet (92). In an AOM/DSS mouse model, 6-week sucralose supplementation (1.5 mg/ml) prior to AOM/DSS treatment increased mucosal occludin, claudin-1, and claudin-4 (vs. mice that were not supplemented) (6).

#### Sweet Taste and Bitterness Receptors in the Gut

Sweet taste receptors are composed of a heterodimer of taste 1 receptor member 2 (T1R2) and taste 1 receptor member 3 (T1R3) (128). AS bind to human and rodent G protein-coupled sweet taste receptors T1R2/T2R3 present in the oropharynx and enteroendicrine cells of the gut and pancreas (129, 130), and to the human bitter taste receptors T2R43 and T2R44 (131). These receptors are involved in nutrient sensing and appetite modulation, glucose homeostasis, and gut motility (132). In the mammalian gut, T1R2 and T1R3 are present in the small intestine and control the release of peptide hormones such as glucagon-like peptide-1 (GLP-1), glucagon, peptide YY (PYY), neuropeptide Y, and cholecystokinin (CCK) (128, 133–139). More recently, T1R3 has also been implicated for its role in modulating epithelial integrity (7). Of note, the activation of toll-like receptors (TLRs) is crucially sensitive to cellular cholesterol, and conversely, TLR activation modulates disposal pathways for cellular cholesterol (124). This is important considering that AS have been shown to increase liver cholesterol and serum low-density lipoprotein (LDL) levels but decrease the level of HDL (2).

Of importance is variants in taste receptor genes, which exist between and within vertebrate species, that result in functional receptor changes or altered expression levels may be associated with metabolic conditions (140). Variants can also affect the perception of sweetness (e.g., aspartame perceived as sweet by humans but not rodents) (141). Even a single amino acid change can affect the functionality of the sweet taste receptor, and in turn, its ability for ligand binding. For instance, a common variant in rodent strains reduces the affinity of the T1R3 subunit for sugars (142, 143).

*Via* binding to receptors, AS regulate various processes such as glucose transport and insulin secretion (144, 145), although findings appear to vary based on mouse line. For instance, in male Sprague Dawley rats, saccharin impaired glucose homeostasis and GLP-1 release (146). In another study, saccharin elicited the most profound effects on glucose intolerance when compared with sucralose or aspartame in C57Bl/6 WT mice (3). In CD1 mice, short- and long-term (6, 12 weeks) stevia supplementation in drinking water (4.16 mg/mL) increased, body weight, insulin, leptin, glycemia, and the secretion of GIP but did not affect food intake (100). In contrast, in Zucker diabetic fatty rats, a rat model of Type 2 diabetes that harbors a missense mutation in the leptin receptor gene, stevia and sucralose had no effect on glucose levels; while in male Wistar rats and B6 mice, stevia, sucralose, and Ace-K supplementation had no effect on plasma GIP-1 or GIP (147). Of note, the key enzyme responsible for aspartame metabolism, aminopeptidase A, is expressed predominantly in the mid to distal sections of the small intestine (148), suggesting that proximal regions of the small gut are indeed exposed to un-metabolized aspartame, which is able to bind to the sweet taste receptor T1R3 expressed in these regions (149). The presence of key taste reception signaling components in different distributions in Paneth cells, which contain amino acid taste receptor components, suggests that Paneth cells have distinct sensing roles, and that they may be involved in the response to AS (145).

Despite *in vitro* reports of T1R3 being key to the observed cellular effects (7, 37), the findings need to be confirmed using *in vivo* permeability models to establish physiological relevance. For example, saccharin supplementation (0.1 mg/mL) in a chemically induced colitis C57BL/6jRj wild-type mouse model elicited no effect on t1r2 and t1r3 expression, or on the taste-receptor-associated neurotransmitter cck or pyy mRNA levels in gut tissue when compared to non-treated mice (20). These differential effects, compared with those reported *in vitro*, may be, in part, due to microbiota change alterations in host cells.

### Tumor Necrosis Factor-Alpha, Toll-Like Receptor, and NF-κB

Toll-like receptor pathways play an important role in activating innate immune response, and its activation by LPS triggers the activation of the NF-κB pathway and the transcription of pro-inflammatory genes such as TNFα (119, 150). NF-κB is a transcription factor downstream of the mitogen-activated protein kinase (MAPK) signaling pathway that induces the production of inflammatory cytokines such as TNFα, IL-1β, and IL-6, as well as the expression of pro-inflammatory enzymes such as cyclooxygenase-2 (COX-2) and inducible nitric-oxide synthase (iNOS), all of which are involved in the pathogenesis of IBD. Enhanced generation of reactive oxygen species (ROS) and reactive nitrogen species (RNS), which can activate metallothionein expression and in turn NF-κB activation, is also reported in the intestine during IBD.

*In vitro* studies suggest that the immunomodulatory effect of steviol glycoside and its related compounds involve NF-κB signaling. These effects however, appear to vary based on the type and dosage of the compound tested. For example, in the human monocytic cell THP-1, stevioside (at 1 mM) suppressed the LPS-induced production of inflammatory mediators TNFα and IL-1β, and mildly suppressed NO release by interfering with the IKKβ and NF-κB signaling pathways, but steviolat 100 μM did not (104). Notably, the administration of stevioside alone, in the absence of LPS, elicited a small increase in TNFα secretion partially mediated through TLR4 (104). In another study using RAW264.7 cells, stevioside was shown to dose-dependently exert anti-inflammatory activity by inhibiting NF-κB activation and MAPK signaling and the expression of TNFα, IL-6, and IL-1β in LPS-stimulated cells (151). Similarly, in another study, stevioside administration resulted in significant reduction of TNFα, nitrates, and ROS production compared with RAW264.7 cells treated with LPS alone (12).

In line with *in vitro* evidence, animal studies demonstrated an anti-inflammatory effect of stevioside. In a mouse model of *Staphylococcus aureus*-induced mastitis, stevioside reduced inflammatory cell infiltration and the expression of TNFα, IL1-β, and IL-6 *via* the TLR2, NF-κB, and MAPK signaling pathways (152). Similarly, in male Balb/c mice, supplementation with stevoside, at either high (100 mg/kg BW) or low (50 mg/kg BW) dose for 7 days prior to DSS colitis induction significantly lowered colonic TNFα levels and attenuated the NF-κB and MAPK signaling pathways (12).

Rodent studies have also reported the increased expression of TLR4, TNFα, and NF-κB following sucralose supplementation (6, 92). In a DSS-colitis C57BL/6 mouse model, sucralose treatment increased the expression of TNFα, TLR4, and Myd88 but decreased the expression of IL-10 and IκBα (6).

### Peroxisome Proliferator-Activated Receptor (PPAR)-Alpha

Peroxisome proliferator-activated receptor (PPAR)-alpha is a ligand-activated transcriptional factor that regulates fatty acid beta oxidation gene expression and is a major regulator of energy homeostasis. PPARα is a predominantly expressed tissue with a high level of fatty acid catabolism such as in liver, heart, muscle, and intestine (153). PPARα agonists have been shown to exert anti-inflammatory and anti-thromobotic activities in both the vascular wall and the liver (154).

In male Wistar rats, sucralose (1.5%) exerted the highest effect on stimulating PPARα expression and CPT-1 compared with other sweeteners (sucrose, fructose, glucose, steviol glycosides, brown sugar, honey, and steviol glycoside+sucrose), and to that of untreated mice (plain water) (92). The effect of sucralose on PPARα expression was suggested to explain the increased formation of ketone bodies and gluconeogenesis, which in turn increased glucose and insulin levels and glucose intolerance, as seen in sucrose-fed mice (92). While PPARα activators have been shown to regulate obesity in rodents, these effects are influenced by estrogen and, thus, are exerted with specific dimorphism (155). In this regard, more studies on both male and female animals are required.

### Cytochrome P450-Xenobiotic Detoxification

In Sprague Dawley rats, Splenda exerted a dose-dependent effect on the expression of intestinal p-glycoprotein and intestinal cytochrome P-450 (CYP), which are involved in xenobiotic detoxification in the gut and the liver. That is, Splenda enhanced the expression level of P-gp at dosages of 300, 500, and 1,000 mg/kg/d, and enhanced CYP3A4 (1,000 mg/kg/d) and CYP2D1 (500 and 1,000 mg/kg/d) expression (56). It is possible that the enhanced expression of P-gp and CYP at higher concentration affects the bioavailability of Splenda; thus, less Splenda is absorbed at higher concentrations, resulting in both more pronounced effects on the gut microbiota and differences in weight.

### Oxidative Stress

Oxidative stress is an important regulator of claudin-3 and is associated with LPS-induced permeability.

Animal studies have reported an antioxidant effect following stevioside treatment (156). In DSS-colitis male Balb/c mice, high and low doses of stevioside (100 and 50 mg/kg body weight) decreased the expression of pro-inflammatory enzymes COX-2 and iNOS, decreased the levels of MPO activity (maker of neutrophil infiltration), and restored the activities/levels of antioxidant enzymes (SOD, CAT, GST, and GSH) in colon tissue when compared with non-supplemented DSS-colitis mice (12). The expression of HO-1, which is an index of cyto-protective and antioxidant enzymes, was also increased in stevioside-treated groups (12).

Saccharin supplementation (0.3 mg/ml, 6 months) in C57BL/6 mice elevated hepatic nitric-oxide synthase (NOS) and TNFα (4). In comparison, sucralose supplementation (0.1 mg/ml, 6 months) elevated the expression of pro-inflammatory mediators in the liver, namely, matrix metalloproteinase 2 (MMP-2) and iNOS (5). Notably, catalase and catalase-peroxidase, two bacterial anti-oxidative genes known to respond to ROS and by themselves can stimulate pro-inflammatory cytokines (157), were also found to be enriched in sucralose-supplemented groups (5).

In male Wistar albino rats, aspartame (40 mg/kg/day, 90 days) significantly increased serum lipid peroxidation and nitric oxide concentrations with concomitant decrease in serum levels of primary scavenging enzymes superoxide dismutase, catalase, glutathione peroxidase, and glutathione (GSH) (158).

### Intracellular Adhesion Molecule (icam-1) and Immunoglobulins

Short-term saccharin supplementation (drinking water, 0.1 mg/ml), either before or after the induction of acute or chronic DSS-induced colitis, significantly decreased the mRNA levels (vs. controls) of colonic intracellular adhesion molecule (*icam-1*), a key regulator of inflammatory signaling, (20) *via* NF-κB activation (159). Saccharin-supplemented mice also exhibited reduced serum protein level of KC, a neutrophil recruiting cytokine that is induced by inflammatory stimuli in immune and epithelial cells (160, 161), suggesting that saccharin lowered the expression of inflammatory markers and did not induce inflammation.

The effect of AS on Immunoglobulins (Ig) has also been examined. Balb/c mice supplemented with either stevia (4.2 mg/ml stevia given as SweetLeaf) or sucralose (5.2 mg/ml sucralose given as Sweetal) for 18 weeks resulted in increased levels of different immunoglobulins (IgG, IgE, and IgA) compared with supplemented controls (received normal drinking water) (2).

### Artificial Alternative Using Sugar-Silica as a Substitute to AS, and Its Effect on Intestinal Inflammation

Sugar has been used as the gold standard for comparing the sweetening efficacy of AS to enable the activation of sweet perception with less calories per unit of food. As an artificial alternative to AS, others have recently proposed to reduce the amount of sugar required to activate the sweet taste receptors in the tongue by combining sugar with silica in special structural reversible binding. With this alternative, proponents suggest that the use of diluted sugar will provide sufficient sweetening power with less molecules of sugar, lowering the caloric content of meals. A logical concern is the uncertainty that exists surrounding the effect of the use of silica on intestinal health and inflammation. Silica is a broad term that encompasses various forms of silicon (Si, a semimetal in the periodic table of elements) compounds that exist in nature. Some forms of silica are used as (i) food additives (silicon oxide, SiO_2_, PubChem ID 5461123, anti-caking powder), (ii) silica gel desiccant to absorb moisture (packages labeled with warning “do not eat”), and (iii) some forms are widely present in nature as crystalline silica (mineral found in sand, stone, concrete, and mortar).

As with other AS, proprietary formulations of sugar-silica as a carrier compound (80, 162) make the interpretation of health effects challenging. Of concern is that the oral consumption of small particles of silica dioxide (<100 nm) has been shown to exacerbate DSS colitis in a size-dependent manner (163). Specifically, daily intake of 10-nm-sized SiO_2_ nanoparticles exacerbated colitis in wild-type C57BL/6J mice, whereas 30-nm nanoparticles had no colitic effect. Mechanistic studies showed that the severity of colitis induced by 10-nm particles was prevented when mice were deficient in apoptosis-associated speck-like protein containing a C-terminal caspase recruitment domain CARD (PYCARD gene, or ASC) (164), indicating that small SiO_2_ nanoparticles aggravate colitis through activation of the PYCARD inflammasome (163). Regarding microbiome composition, summary indicators of diversity at the genus level (principal component analysis and rarefaction curves based on chao1 index) were reported to be similar between the SiO_2_-fed and control mice (at 3 mg/kg/day); however, no complete data of microbiome analysis were presented to infer the effect in detail. With aggravating changes in inflammasome and colitis, gut microbiome alterations are expected, as shown for AS. On metabolism, studies have shown that the metabolism of Si is complex depending on the size of the molecule and may have various effects on health. Once ingested, silicon reacts systemically with other molecules such as those of trace elements (zinc, copper, iron) (165) and lipids in the gut and blood (166). Taken together, SiO_2_ should be used cautiously given the potential impact on human health.

## Conclusions

Overall, the data illustrate that the effect of AS on inflammation is multifactorial and depends on various factors such as dosage, type of compound, and host genetics. Of increasing relevance to patients with chronic digestive inflammatory disorders, evidence suggests that AS may induce pro-inflammatory changes in gut bacteria and gut wall immune reactivity, which could negatively affect individuals with or susceptible to chronic inflammatory conditions. However, there is a need to further reproduce these findings in various mouse lines, across different diets, and different artificial sweeteners in the context of the fillers used in commercial products (e.g., maltodextrin).

Studying the causal effect of individual dietary ingredients (e.g., AS) is challenging, given that most commercial products contain a commercial proprietary blend of two or more AS, as well as ingredients, to add weight and volume and/or make AS more palatable. Nevertheless, findings from commercial products are desirable and may be more readily translatable to humans.

## Author Contributions

AB, AR-P, and FC: study design, manuscript writing, review, comments, and editing of final manuscript. AB and AR-P: literature review. All authors contributed to the article and approved the submitted version.

## Funding

This publication was supported by NIH grants DK42991, DK055812, DK091222, and DK097948 (to FC), DK118373 and AI143821 (to AR-P), DK127008 (to AB), and a grant from the NIH Cleveland Digestive Diseases Research Core Center (DDRCC) Administrative Core (to FC).

## Conflict of Interest

The authors declare that the research was conducted in the absence of any commercial or financial relationships that could be construed as a potential conflict of interest.

## Publisher's Note

All claims expressed in this article are solely those of the authors and do not necessarily represent those of their affiliated organizations, or those of the publisher, the editors and the reviewers. Any product that may be evaluated in this article, or claim that may be made by its manufacturer, is not guaranteed or endorsed by the publisher.
